# Lattice Rayleigh Anomaly Associated Enhancement of NH and CH Stretching Modes on Gold Metasurfaces for Overtone Detection

**DOI:** 10.3390/nano10071265

**Published:** 2020-06-29

**Authors:** Daler R. Dadadzhanov, Tigran A. Vartanyan, Alina Karabchevsky

**Affiliations:** 1 School of Photonics, Center of Information Optical Technology, ITMO University, 49 Kronverksky pr., St. Petersburg 197101, Russia; tigran.vartanyan@mail.ru; 2 School of Electrical and Computer Engineering, Ben-Gurion University of the Negev, Beer-Sheva 8410501, Israel

**Keywords:** metasurface, localized surface plasmon resonance, surface-enhanced near-infrared absorption, overtone spectroscopy

## Abstract

Molecular overtones stretching modes that occupy the near-infrared (NIR) are weak compared to the fundamental vibrations. Here we report on the enhancement of absorption by molecular vibrations overtones via electromagnetic field enhancement of plasmonic nanoparallelepipeds comprising a square lattice. We explore numerically, using finite element method (FEM), gold metasurfaces on a transparent dielectric substrate covered by weakly absorbing analyte supporting N-H and C-H overtone absorption bands around 1.5 μm and around 1.67 μm, respectively. We found that the absorption enhancement in N-H overtone transition can be increased up to the factor of 22.5 due to the combination of localized surface plasmon resonance in prolonged nanoparticles and lattice Rayleigh anomaly. Our approach may be extended for sensitive identification of other functional group overtone transitions in the near-infrared spectral range.

## 1. Introduction

Near-infrared (NIR) spectroscopy is a powerful method of non-destructive material analysis based on the excitation of overtones and combination bands of molecular vibrations [1,2,3,4]. An important advantage of NIR spectroscopy over the spectroscopes in the longer wavelengths is the availability of effective radiation sources and sensitive detectors [5,6]. On the other hand, NIR transitions, being forbidden in the harmonic approximation, are much weaker than the transitions corresponding to the fundamental vibration modes [7,8]. Hence, to be applicable in small sample analysis, NIR absorption should be substantially enhanced. Due to this fact, searching for the means of NIR absorption amplification has recently become the focus of many studies [4,9,10,11,12,13,14,15,16].

Localized surface plasmon resonance (LSPR) excited in metal nanoparticles has been known to provide substantial absorption enhancement by nearby molecules [17,18,19]. Previously, we have shown that the size and the aspect ratio of prolate gold nanospheroids may be chosen in such a way that their LSPR bands overlap with the first-order overtones of C-H and N-H stretching modes of organic substances [20]. Next, the size and the shape of a rod-like gold nanoparticle was optimized to achieve the maximum overtone absorption enhancement [21]. However, using the isolated gold nanoparticles implies that only a very small fraction of the analyte is placed in the amplified near-field. This circumstance limits the overall enhancement provided by LSPR. To get the most of the plasmon field amplification, metal nanoparticles are to be arranged in a dense array forming a metasurface. Consequently, the larger portion of the analyte molecules may be placed in the regions of enhanced near-field [22]. Moreover, an electromagnetic interaction between the adjacent nanoparticles leads to an additional mechanism of local field enhancement when the conditions for the Rayleigh anomaly are fulfilled [23,24,25,26,27,28,29,30,31,32]. Fabrication of nanoparticle arrays of required density is feasible via electron beam lithography (EBL) [33,34], although the shapes of the nanoparticles defined by EBL on a substrate are closer to the nanoscale parallelepipeds (NPs) rather than to the nanorods or nanospheroids.

Therefore, this study is devoted to planar metasurfaces consisting of nanoparallelepipeds (NPs) that can be fabricated easily and prepared specifically for overtone detection in near-infrared. The proposed structure utilizes a combination of localized plasmon resonance in isolated gold NP with Rayleigh anomalies of their periodic array. By incorporating the plasmonic metasurface into a weakly absorbing organic medium, a 22.5-fold enhancement of the first overtone of N-H stretching mode in the NIR has been demonstrated for the first time. Application of the designed gold metasurface may be beneficial in biomedicine [35], non-destructive testing [36], and food quality analysis [37,38,39].

## 2. Numerical Model

**Figure 1:**
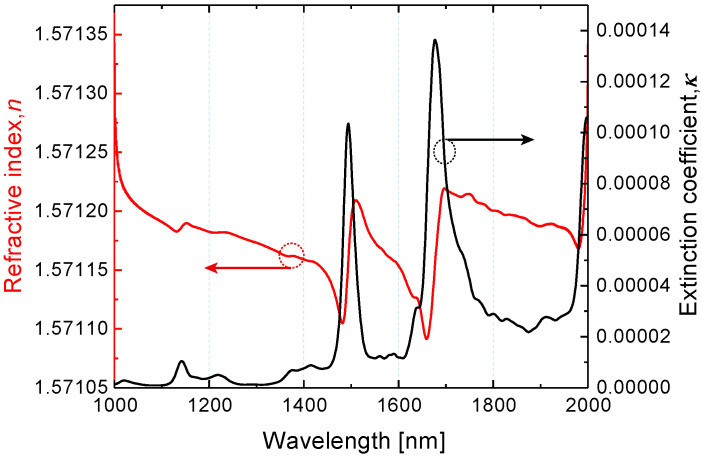
Dispersion characteristic of the N-Methylaniline (NMA) molecules as a function of the wavelength in the near-infrared.

To obtain a realistic model we consider an infinite array of gold nanoantennas in the form of parallelepipeds arranged in a square lattice on a dielectric substrate and covered by a thin layer of analyte molecules. To be specific, as a probe molecule, we work with the well-known organic compound and derivative of Ammonia, N-Methylaniline (C_6_$H_{5}NH\left(CH_{3}\right))$ [9,40]. Recently, we have thoroughly studied the optical properties of N-Methylaniline in bulk samples and, using Kramers–Kronig relations, obtained its complex dielectric permittivity in the NIR [4,9,10,41]. The absorption bands (Figure 1) at wavelengths of 1494 and 1676 nm are associated with the first overtones of N-H and C-H stretching modes and accompanied by the anomalous dispersion.

**Figure 2:**
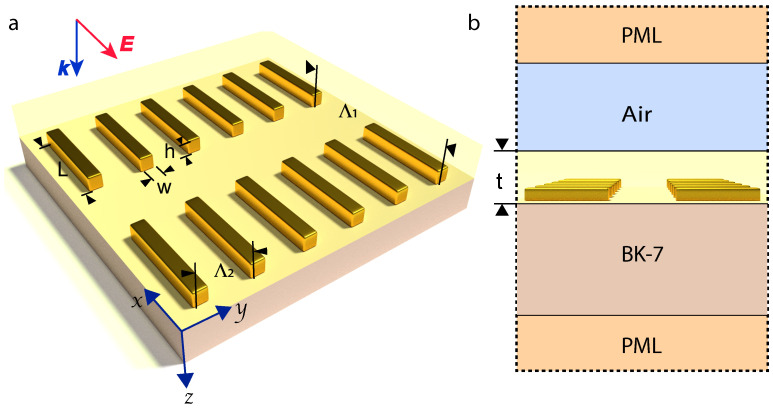
(**a**) 3D schematics of the gold metasurface modeled in COMSOL Multiphysics software. The width and height of gold nanoparallelepipeds (NPs) in the array are of w=h = 20 nm, while the length is varied. The longitudinal and transverse lattice constants are defined as $\Lambda_{1}$ and $\Lambda_{2}$. *t* designates the analyte film thickness. The incident light propagation direction *k* and polarization *E* are also shown. (**b**) Schematics of 2D cross-section of the model. Furthermore, the gold metasurface submerged into the analyte layer, the structure involves a BK7 glass substrate and an air layer with refractive index *n* = 1. The sample is placed between perfectly matched layers (PML).

Numerical simulations via finite-element-method (FEM) were conducted in the COMSOL Multiphysics environment. The capabilities of the software allow us to calculate plasmonic properties of gold metasurface of studied geometry with high accuracy based on a straightforward solution of Maxwell’s equations under certain boundary conditions. Here, perfectly matching layers are used to absorb reflected and transmitted light from the sample when the linearly polarized wave ($E_{x}$) is launched perpendicular to the x−y plane through the excitation port placed immediately under the top of a perfectly matched layer. This model also comprises an air gap, as well as the analyte layer and a transparent BK7 glass substrate (see Figure 2). A 2D periodic array of NPs illustrated in Figure 2a was modeled using the Floquet type boundary conditions that were imposed on the side walls of an elementary cell shown in Figure 2b. More details on the model description are given in [22]. Thus, the gold metasurface is modeled as a square lattice of prolonged NPs with variable length *L*, while width *w* and height *h* were fixed and equal to 20 nm (depicted in Figure 2a,b). The dispersion of dielectric properties of gold was obtained from the experimental work of Johnson and Christy for bulk material [42] without modification because the nanoparticles dimensions are large enough for the possible changes to be insignificant in our case [43,44]. Dispersion of BK7 used as a substrate was taken from [45].

Since we used the periodic boundary conditions, the results are strictly valid for the infinite structures, while all the real structures are finite. There are several studies devoted to the relation between the optical properties of finite structures of different sizes and the corresponding infinite structure [46,47,48]. Although there is no general solution, in most cases several hundreds of periods are enough for the convergence.

To facilitate the comparison of the obtained theoretical results with the future experimental data, we introduce the notion of ’*Differential transmission*’, which helps to explore the effect of the gold metasurface on the overtone absorption intensity. The differential transmission is computed as a difference between transmissions of analyte and immersion oil films of the same thickness. Related notions of differential extinction and differential absorption were previously introduced in [21,41]. A transparent dielectric film characterized by a negligible dispersion was used as a reference for the differential transmission calculations. The refractive index of this film was chosen to be the same as the mean value of the N-Methylaniline (NMA) refractive index in the actual spectral range ($n_{av}$ = 1.5712). Experimentally, such a film may be readily realized utilizing an appropriate immersion oil. Transmission spectra of plane parallel films without gold nanoparticles were calculated analytically [49].

## 3. Optimization of the Metasurface

**Figure 3:**
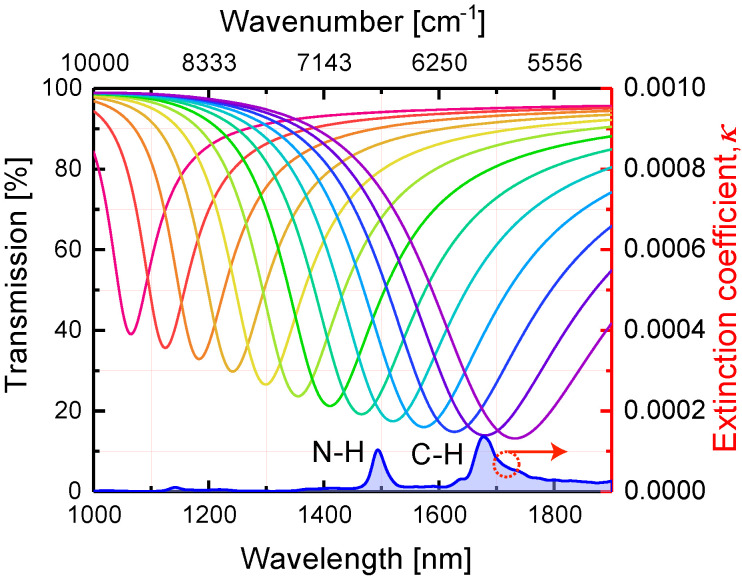
Calculated transmission spectra of gold metasurfaces with NPs of lengths *L* varied from 100 (crimson curve) to 220 (purple curve) nm in 10 nm steps, whereas lattice periods were fixed at $\Lambda_{1}$ = 400 nm and $\Lambda_{2}$ = 200 nm. The extinction coefficient of NMA is shown below the transmission curves. The gold metasurface was embedded in NMA with a thickness of 100 nm.

**Figure A1:**
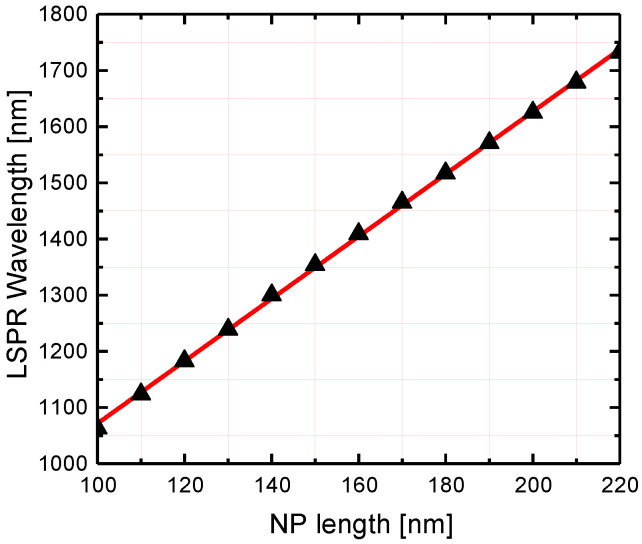
The LSPR spectral position as a function of NPs length *L*. The metasurface parameters were set to the following values: w=h = 20 nm, *t* = 100 nm, $\Lambda_{1}$ = 400 nm, and $\Lambda_{2}$ = 200 nm.

When the lattice periods become comparable to the sizes of the individual nanoantennas, plasmon resonances start to interact with the lattice resonances. Hence, the optimization procedure becomes very complex. To facilitate the optimization, we study the Rayleigh anomaly that corresponds to the first diffraction order which starts to propagate into the substrate with the refractive index n=1.5 at the wavelength of N-H overtone λ=1494 nm (in vacuum). The rough approximation of the lattice constant is $\Lambda_{1}=\lambda /n$, as shown in Figure 2a, gives a value of 1 μm. Next, the length of the nanoantenna is to be chosen in such a way that the effective polarizability of the nanoantennas comprising the metasurface $\alpha^{*}$ reaches its maximum value. According to the well-developed theory of diffractively coupled localized plasmon resonances in the framework of the coupled dipole approximation (CDA) [24], $\alpha^{*}$ may be calculated as follows:

(1) $$\alpha^{*}=\frac{1}{\frac{1}{\alpha }-S}$$

where α denotes the polarizability of an isolated nanoantenna that depends solely on its size and shape (at the fixed wavelength). The dipole sum *S* accounts for the dipole-dipole interaction between nanoantennas and only depends on the lattice periods $\Lambda_{1}$, $\Lambda_{2}$ (see Figure 2a). According to Equation (1) $\alpha^{*}$ reaches its maximum when the real parts of *S* and $\alpha^{-1}$ are equal. From the calculated results presented in [24], we learn that *S* is very small anywhere but at the narrow window around $\frac{\Lambda_{2}}{\lambda }\approx 1$ it has a maximum. On the other hand, the LSPR wavelength of NP of a fixed cross-section is a monotonic function of its length as it may be seen in Figure 3 and Figure A1. Hence, to maximize the effective polarizability at the wavelength of the molecular overtone transition, the length of the nanoantenna should vary together with the lattice periods. The length of the resonance antenna is expected to have a minimum as a function of the lattice period. Considering this, we performed the numerical simulation and found the structures of gold metasurfaces with certain LSPR bands optimized for sensing of functional groups overtone transitions at λ=1494 nm and λ=1676 nm.

## 4. Dependence of the Collective LSPR Spectral Position on the Gold Metasurface Parameters

The dependence of the transmission dip location on the nanoparticle length has been investigated. We performed the simulations for the light polarized along the length of gold NPs (*x*-axis) at normal incidence. Figure 3 shows the transmission spectra of gold metasurfaces with fixed lattice periods and varying lengths of nanoantennas. In close analogy to the well-known dependence of the LSPR spectral position on the aspect ratio of an isolated prolate spheroid, the dip in transmission shifts toward the long-wavelength range when the NPs length *L* grows. At particular values of length *L* the transmission dip is defined by collective LSPR overlaps with and couples to the N-H and C-H overtone absorption bands in near-infrared. The transmission dip location depends linearly on the NPs length *L*, as can be seen in Figure A1.

## 5. Near-Field Enhancement

**Figure 4:**
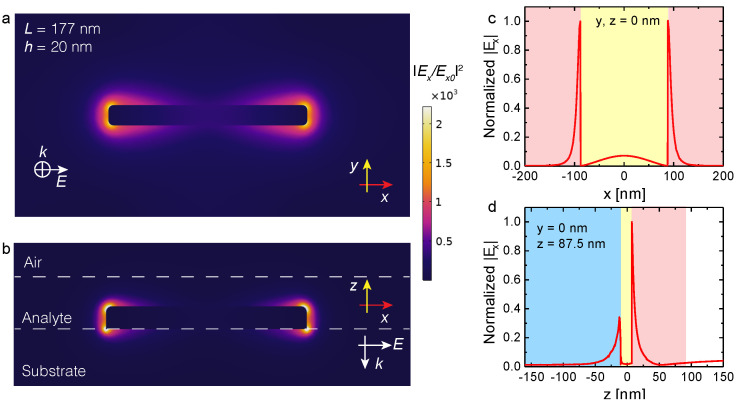
The electric field enhancement ${\left|E_{x}/E_{x0}\right|}^{2}$ distribution around the gold NP in an array ($\Lambda_{1}$ = 400 nm and $\Lambda_{2}$ = 200 nm) surrounded by NMA layer: top view (**a**) and side view (**b**). Normalized electric field distribution along the *x*-axis goes through the NP center (**c**); the same for the *z*-axis (**d**) at *x* = 87.5 nm. Color encoding in (**c**,**d**): blue—the BK-7 glass substrate, yellow—gold nanoparticle, red—thin layer of NMA layer, white—air. The excitation wavelength is λ = 1494 nm. The colorbar corresponds for colormaps in (**a**,**b**) subplots.

In this section, we analyze the distribution of the enhanced near-field around the gold NP. The incident field is polarized along the *x*-axis while the lattice constants are fixed at $\Lambda_{1}$ = 400 nm and $\Lambda_{2}$ = 200 nm, respectively. Figure 4 shows the near-field distribution around one of the gold nanoantennas in the array when the collective LSPR of the metasurface is tuned to coincide with one of the analyte overtones at 1494 nm. The top (a) and side (b) views are both shown in Figure 4. Electric near-field polarized along the long axis of the NP exhibits strong enhancement and localization around the antenna tips. Figure 4c,d show the corresponding field distributions along the *x* and *z* axes. We note that the calculated results shown in Figure 4 demonstrate the lightning-rod effect (subplots a, b) as well as the rapid near-field decay (subplots c, d) inside the homogeneous layer of organic molecules and the dielectric substrate. Indeed, the length of NP is more than eight times larger than its width and height. Hence, the main prerequisite for the field concentration at the sharp edges of the nanoparticle and the lightning-rod effect observation is fulfilled. Large jumps of the electric field at the metal surfaces seen in Figure 4 is due to the large value of the real part of gold permittivity at λ = 1494 nm (about minus one hundred).

## 6. Differential Transmission Computations

**Figure 5:**
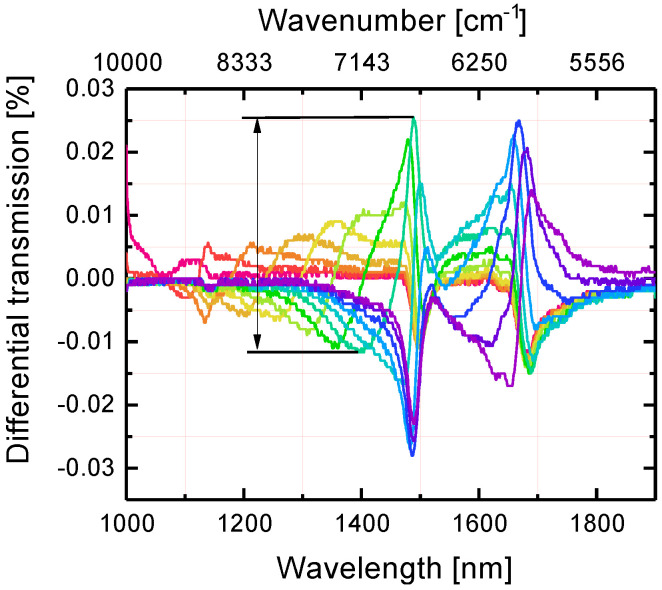
Differential transmission (DT) of metasurfaces with 100 nm thick analyte overlayers. The length of NPs varies from 100 (crimson curve) to 220 (purple curve) nm with steps of 10 nm while the lattice periods are of $\Lambda_{1}$ = 400 nm and $\Lambda_{2}$ = 200 nm. The DT contrast is defined as the difference between the maximum and minimum values of DT in the spectral range of the N-H overtone transition and is shown by an arrow for one of the metasurfaces.

**Figure A2:**
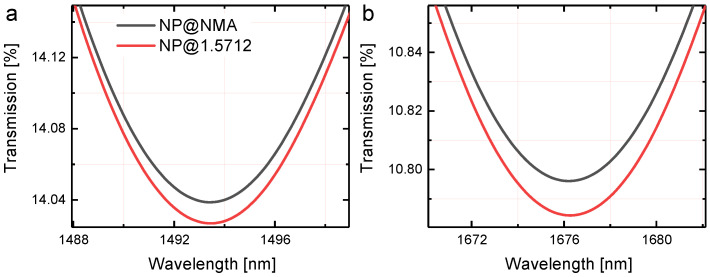
A closer look at the transmission spectra of metasurfaces covered by the NMA layer (black curves) and immersion oil with $n_{av}=1.5712$ (red curves) of the same thickness t=35 nm. The NPs lengths *L* were chosen to provide for the coincidence of the collective LSPRs of the metasurfaces with the NMA overtone transitions: (**a**) L=196 nm for N-H band and (**b**) L=234 nm for C-H band.

Computation of the differential transmission (DT) spectra is an important step in metasurface design for sensing purposes where the crucial feature is the detection of a small amount of analyte. In particular, the enhanced absorption of the analyte molecules in the near-field of the metasurface is still much smaller than the own absorption of the metasurface in the absence of molecules. In this regard, to reveal the contribution of the gold metasurface, the NPs absorption excluding the presence of molecules must be subtracted from the measured absorption with molecules. Furthermore, it is important to take into account that the metasurface absorption spectra shift when the permittivity of surrounding changes. Therefore, measurement of the metasurface transmission in air seems infeasible. Instead, the metasurface transmission in contact with a thin film of a transparent material should be considered. The refractive index (RI) of this material should be chosen close to the RI mean value of analyte in the actual spectral range and the film thickness should be the same as that of an analyte. Based on that, calculation of DT becomes a reliable way to reveal the presence of analyte and the spectral position of its absorption bands [22]. Figure 5 displays an example of the DT spectrum. Compared to what is shown in the literature [50,51], the plasmon enhanced absorption DT spectrum demonstrates complex behavior (see Figure 5). DT changes its sign as a result of combined action of absorption and anomalous dispersion of the analyte in the spectral ranges of overtone transitions. The regions of enhanced and reduced transmission alternate. Figure A2a,b demonstrate the spectral regions where the counterintuitive relation between transmission of the metasurface embedded in NMA and transmission of the same metasurface embedded in immersion oil takes place. The structure covered by a dispersive and absorptive NMA film transmits more light than the same structure embedded in the transparent and dispersionless immersion oil film.

**Figure 6:**
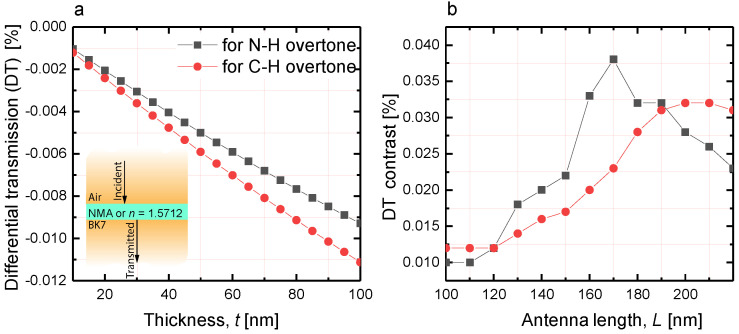
(**a**) DT of NMA films on a bare substrate as function of the film thickness. The insert shows the structure of the parallel films used for the calculation under the normal incidence illumination. (**b**) The DT contrast estimated from the data presented in Figure 5 as a function of the nanoantenna length.

For the sensing purposes, the difference between the DT maximum and minimum (hereinafter referred to as ’*DT contrast*’) is of paramount importance, since it is the variation magnitude that determines the metasurface sensitivity. Then, the enhancement factor (EF) can be expressed as $EF=\frac{DT}{DT_{0}}$, where $DT_{0}$ is the DT value of the same analyte film placed on a bare substrate without gold metasurface. $DT_{0}$ values were obtained via elementary calculations and presented in Figure 6a for a range of analyte film thicknesses up to *t* = 100 nm. Figure 6b shows the DT contrasts for the arrays of nanoantennas of different lengths presented in Figure 5. We note that the EF provided by metasurface utilization depends on the nanoantennas lengths. Hence, the optimization procedure is essential to maximize the EF for the particular overtone transition.

## 7. Enhancement Factors of the Optimized Metasurfaces

**Figure 7:**
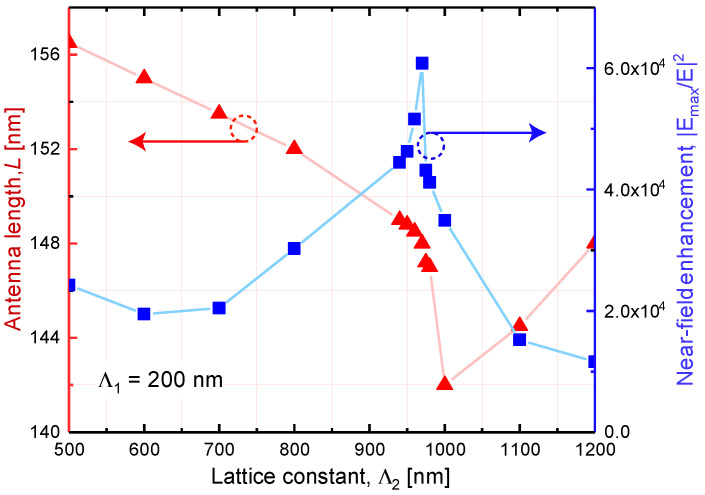
Variation of the local field enhancement (blue curve) and the resonant nanoantenna length *L* (red curve) with the lattice period $\Lambda_{2}$ when the other lattice period is fixed at $\Lambda_{1}$ = 200 nm and ensuring constant collective Localized surface plasmon resonance (LSPR) band at λ = 1494 nm. The analyte film thickness *t* = 35 nm.

**Figure 8:**
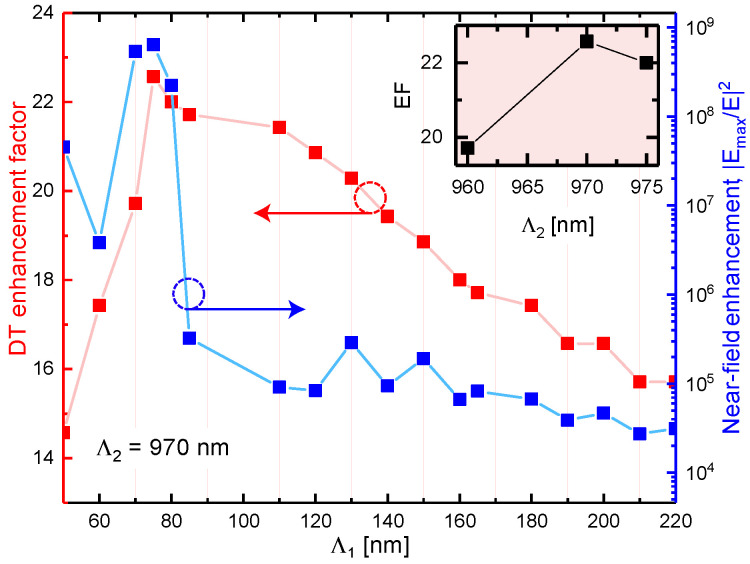
Variation of the local field enhancement (blue curve) and the DT enhancement factor (EF) with the lattice period $\Lambda_{1}$ when the other lattice period is fixed at $\Lambda_{2}$ = 970 nm. The inset shows EF against the lattice constant $\Lambda_{2}$ at fixed $\Lambda_{1}$ = 75 nm. Additionally, the direction and color of the horizontal arrow indicate the corresponding axis for the red and blue curves.

In this section, we discuss how the DT enhancement factor depends on the lattice periods. While exploring the metasurface configuration optimized for the enhancement of the particular overtone transition, the nanoantenna lengths as well as both periods of the square lattice were varied. To reach the maximum field enhancement, the nanoantenna length that maximizes the collective polarizability at the desired wavelength was found for a number of lattice periods combinations. At the final step, the enhancement factors were calculated as presented in Figure 7 and Figure 8. The analyte film thickness was set to 35 nm. This choice provides reasonable utilization of the near-field enhancement region. Indeed, according to Figure 4d, the maximum field enhancement is achieved at the surface of the nanoparticle and rapidly drops with the distance. Hence, from the perspective of the sensitivity enhancement, the analyte layers should not be thicker than 35 nm.

The red curve in Figure 7 shows the variation of the resonant nanoantenna length *L* with the lattice period $\Lambda_{2}$ when the other lattice period is fixed at $\Lambda_{1}$ = 200 nm and ensuring constant collective LSPR band at λ = 1494 nm. In agreement with the numerical results presented in Figure 7, *L* reaches a minimum near the wavelength corresponding to the Rayleigh anomaly at about 1000 nm. Almost simultaneously, at $\Lambda_{2}$ = 970 nm the near-field enhancement reaches its maximum (blue curve in Figure 7). Qualitatively, this behavior may be understood as follows: if we assume that $\Lambda_{2}$ > 996 nm, then the first diffraction order of the radiation with the wavelength λ of 1494 nm (in vacuum) can propagate in the substrate with refractive index $n_{sub}$ = 1.5. However, when $\Lambda_{2}$ < 996 nm, all diffraction orders becomes evanescent since $\Lambda_{2}<\lambda n_{sub}$. The evanescent wave decays fast from the boundary. In addition, it carries no energy. Therefore, its amplitude may exceed the incident wave amplitude without violation of energy conservation law. This is the origin of the lattice contribution to the local field enhancement.

**Figure A3:**
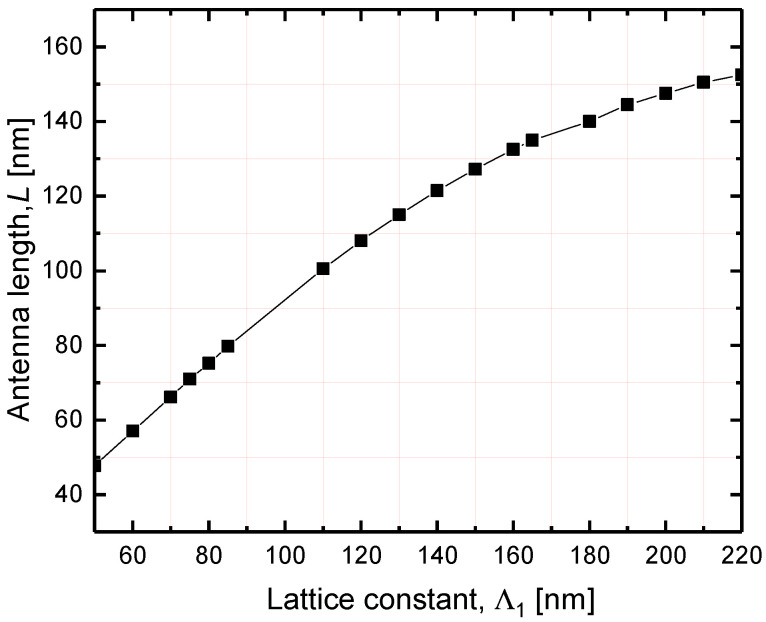
Dependence of the resonance antenna length *L* on the longitudinal lattice period $\Lambda_{1}$ at the fixed $\Lambda_{2}$ = 970 nm.

Subsequently, we fixed $\Lambda_{2}$ at 970 nm and adjusted the second lattice period $\Lambda_{1}$ varying it from 50 to 220 nm, as it is illustrated in Figure 8 (blue curve). Simultaneously with $\Lambda_{1}$ variation, the NPs length *L* was adjusted to keep the collective LSPR of the metasurface at the resonance with the overtone transition in NMA, as it is illustrated in Figure A3. The absolute maximum was found at $\Lambda_{1}$ = 75 nm that corresponds to the field enhancement of 2 ×$10^{8}$. Finally, we checked that this metasurface design also provides for the largest enhancement factor for sensing application which reaches an unprecedented value of 22.5 (red curve, Figure 8). To double check that the absolute maximum of the DT enhancement factor is evaluated, we varied again the $\Lambda_{2}$ around the value of 970 nm. The inset of Figure 8 supports the conclusion that the absolute maximum is found.

## 8. Conclusions

In summary, we have numerically demonstrated the sensing capabilities of the rectangular lattice of gold nanoparallelepipeds on a transparent substrate while tuned on the specific transitions in the near-infrared. The shape of the metasurface unit-cells was adjusted to be readily manufacturable by electron beam lithography or focused ion beam milling. To optimize the metasurface for a particular overtone transition registration, we varied the periods of the lattice and the elements lengths simultaneously. We found that one of the optimized lattice periods is very close to that corresponding to the Rayleigh anomaly. As we are trying to anticipate the response of the actual applications, we do not place the analyte within the near-field enhancement factor maxima, but rather, as a 35 nm thick homogeneous film covering the whole metasurface. Because of that, relatively small proportion of the analyte experiences the largest field enhancement. Consequently, the structure optimized for the sensing of N-H overtone transition at 1494 nm has shown the differential transmission enhancement factor of 22.5. This rather large enhancement may be explained by the concerted action of the LSPR, the lattice resonance and the high surface density of gold nanoparticles on the substrate. Thus, we have demonstrated the way to optimize the gold metasurface for sensing the weak overtone transitions in the near-infrared. This approach may be extended for sensitive registration of other functional group overtones in the near-infrared by tailoring the lattice periods and the aspect ratios of a metasurface unit-cells.

To conclude, we leverage the rapid spectral variation of the refractive index in the spectral range of the analyte anomalous dispersion. As the anomalous dispersion is associated with its overtone absorption band, by measuring the differential transmission of a metasurface tuned to the specified spectral range the tiny amount of an analyte may be detected. In practice, several arrays of nanoparallelepipeds tuned to different spectral ranges may be fabricated on a single chip [1]. In this case, the combination of the responses of all arrays in the chip will lead to the recognition and characterization of the analyte.

## Abbreviations

The following abbreviations are used in this manuscript:

NIR	Near-infrared
LSPR	Localized surface plasmon resonance
EBL	Electron beam lithography
NPs	Nanoparallelepipeds
NMA	N-Methylaniline
DT	Differential transmission
RI	Refractive index

## Appendix A

Figure A1 shows the dependence of LSPR spectral position as a function of NPs length. The gold metasurface was covered by a 100 nm-thick NMA film. The long-wavelength shift of LSPR in longer nanoparticles corresponds to the known from the quasistatics theory behavior of LSPR in prolate particles.

Figure A2 clarifies the origin of the negative values obtained for the differential transmission. Transmission of the NMA covered metasurface is larger than that of immersion oil covered metasurface. Transmission spectra in (a) and (b) were computed for the NPs lengths optimized for sensing of the corresponding overtone transitions.

To keep the collective LSPR of a metasurface in resonance with the chosen overtone transition of NMA, the length of the NPs *L* must be adjusted correspondently. Figure A3 illustrates how the length of the resonant antenna *L* depends on the lattice period $\Lambda_{1}$. As $\Lambda_{1}\ll \lambda$, the Rayleigh anomaly is far away. Because of that, the resonance *L* depends on the inter-particle distance in the predictable from quasistatic arguments monotonic way.

## References

[1] Karbchevsky A., Katiyi A., Ang A.S., Hazan A. (2020). On-chip nanophotonics and future challenges. Nanophotonics.

[2] Blanco M., Villarroya I. (2002). NIR spectroscopy: A rapid-response analytical tool. TrAC Trends Anal. Chem..

[3] Siesler H.W., Ozaki Y., Kawata S., Heise H.M. (2008). Near-Infrared Spectroscopy: Principles, Instruments, Applications.

[4] Katiyi A., Karabchevsky A. Deflected Talbot mediated overtone spectroscopy in near-infrared as a label-free sensor on a chip. ACS Sens..

[5] Ozaki Y., Genkawa T., Futami Y. (2017). Near-Infrared Spectroscopy.

[6] Türker-Kaya S., Huck C.W. (2017). A review of mid-infrared and near-infrared imaging: principles, concepts and applications in plant tissue analysis. Molecules.

[7] Ozaki Y. (2012). Near-infrared spectroscopy—Its versatility in analytical chemistry. Anal. Sci..

[8] Atkins P.W., Friedman R.S. (2011). Molecular Quantum Mechanics.

[9] Karabchevsky A., Kavokin A. (2016). Giant absorption of light by molecular vibrations on a chip. Sci. Rep..

[10] Katiyi A., Karabchevsky A. (2017). Figure of merit of all-dielectric waveguide structures for absorption overtone spectroscopy. J. Lightwave Technol..

[11] Katiyi A., Zorea J., Halstuch A., Elkabets M., Karabchevsky A. (2020). Surface roughness-induced absorption acts as an ovarian cancer cells growth sensor-monitor. Biosens. Bioelectron..

[12] Katiyi A., Karabchevsky A. (2018). Si nanostrip optical waveguide for on-chip broadband molecular overtone spectroscopy in near-infrared. ACS Sens..

[13] Ray G.J., Anderson T.N., Caton J.A., Lucht R.P., Walther T. (2001). OH sensor based on ultraviolet, continuous-wave absorption spectroscopy utilizing a frequency-quadrupled, fiber-amplified external-cavity diode laser. Opt. Lett..

[14] Arsad N., Li M., Stewart G., Johnstone W. (2010). Intra-cavity spectroscopy using amplified spontaneous emission in fiber lasers. J. Lightwave Technol..

[15] Karabchevsky A., Katiyi A., Bin Abdul Khudus M.I.M., Kavokin A.V. (2018). Tuning the Near-Infrared Absorption of Aromatic Amines on Tapered Fibers Sculptured with Gold Nanoparticles. ACS Photonics.

[16] Borovkova O., Ignatyeva D., Sekatskii S., Karabchevsky A., Belotelov V. (2020). High-Q surface electromagnetic wave resonance excitation in magnetophotonic crystals for supersensitive detection of weak light absorption in the near-infrared. Photonics Res..

[17] Klimov V. (2014). Nanoplasmonics.

[18] Chen H., Shao L., Li Q., Wang J. (2013). Gold nanorods and their plasmonic properties. Chem. Soc. Rev..

[19] Giannini V., Fernández-Domínguez A.I., Heck S.C., Maier S.A. (2011). Plasmonic nanoantennas: fundamentals and their use in controlling the radiative properties of nanoemitters. Chem. Rev..

[20] Dadadzhanov D.R., Vartanyan T.A., Karabchevsky A. (2018). Vibrational overtones spectroscopy enabled by plasmonic nanoantennas. Plasmonics: Design, Materials, Fabrication, Characterization, and Applications XVI.

[21] Dadadzhanov D.R., Vartanyan T.A., Karabchevsky A. (2019). Differential extinction of vibrational molecular overtone transitions with gold nanorods and its role in surface enhanced near-IR absorption (SENIRA). Opt. Express.

[22] Dadadzhanov D.R., Vartanyan T.A., Dadadzhanova A.I., Karabchevsky A. (2020). Surface-enhanced near-infrared absorption (SENIRA) of CH and NH groups with gold nanoarray. Quantum Sensing and Nano Electronics and Photonics XVII.

[23] Humphrey A., Barnes W. (2016). Plasmonic surface lattice resonances in arrays of metallic nanoparticle dimers. J. Optics.

[24] Kravets V.G., Kabashin A.V., Barnes W.L., Grigorenko A.N. (2018). Plasmonic surface lattice resonances: A review of properties and applications. Chem. Rev..

[25] Kravets V., Schedin F., Kabashin A., Grigorenko A. (2010). Sensitivity of collective plasmon modes of gold nanoresonators to local environment. Opt. Lett..

[26] Auguié B., Barnes W.L. (2008). Collective resonances in gold nanoparticle arrays. Phys. Rev. Lett..

[27] Chen P.Y., Muljarov E.A., Sivan Y. (2020). Lightning-fast solution of scattering problems in nanophotonics: An effortless modal approach (Conference Presentation). Nanophotonics VIII.

[28] Norman J.C., DeJarnette D.F., Roper D.K. (2014). Polylogarithm-based computation of Fano resonance in arrayed dipole scatterers. J. Phys. Chem. C.

[29] Kanipe K.N., Chidester P.P., Stucky G.D., Meinhart C.D., Moskovits M. (2017). Properly structured, any metal can produce intense surface enhanced Raman spectra. J. Phys. Chem. C.

[30] Wang D., Guan J., Hu J., Bourgeois M.R., Odom T.W. (2019). Manipulating light–matter interactions in plasmonic nanoparticle lattices. Acc. Chem. Res..

[31] Cherqui C., Bourgeois M.R., Wang D., Schatz G.C. (2019). Plasmonic Surface Lattice Resonances: Theory and Computation. Acc. Chem. Res..

[32] Manjavacas A., Zundel L., Sanders S. (2019). Analysis of the Limits of the Near-Field Produced by Nanoparticle Arrays. ACS Nano.

[33] Yokota Y., Ueno K., Mizeikis V., Juodkazis S., Sasaki K., Misawa H. (2007). Optical characterization of plasmonic metallic nanostructures fabricated by high-resolution lithography. J. Nanophotonics.

[34] Grand J., Adam P.M., Grimault A.S., Vial A., De La Chapelle M.L., Bijeon J.L., Kostcheev S., Royer P. (2006). Optical extinction spectroscopy of oblate, prolate and ellipsoid shaped gold nanoparticles: experiments and theory. Plasmonics.

[35] Luypaert J., Massart D., Vander Heyden Y. (2007). Near-infrared spectroscopy applications in pharmaceutical analysis. Talanta.

[36] Nicolai B.M., Beullens K., Bobelyn E., Peirs A., Saeys W., Theron K.I., Lammertyn J. (2007). Nondestructive measurement of fruit and vegetable quality by means of NIR spectroscopy: A review. Postharvest Biol. Technol..

[37] Prieto N., Pawluczyk O., Dugan M.E.R., Aalhus J.L. (2017). A review of the principles and applications of near-infrared spectroscopy to characterize meat, fat, and meat products. Appl. Spectrosc..

[38] Dos Santos C.A.T., Lopo M., Páscoa R.N., Lopes J.A. (2013). A review on the applications of portable near-infrared spectrometers in the agro-food industry. Appl. Spectrosc..

[39] Xia Y., Xu Y., Li J., Zhang C., Fan S. (2019). Recent advances in emerging techniques for non-destructive detection of seed viability: A review. Artif. Intell. Agric..

[40] Shaji S., Eappen S.M., Rasheed T., Nair K. (2004). NIR vibrational overtone spectra of N-methylaniline, N, N-dimethylaniline and N, N-diethylaniline—A conformational structural analysis using local mode model. Spectrochim. Acta Part A Mol. Biomol. Spectrosc..

[41] Karabchevsky A., Shalabney A. (2016). Strong interaction of molecular vibrational overtones with near-guided surface plasmon polariton. Optical Sensing and Detection IV.

[42] Johnson P.B., Christy R.W. (1972). Optical constants of the noble metals. Phys. Rev. B.

[43] Stietz F., Bosbach J., Wenzel T., Vartanyan T., Goldmann A., Träger F. (2000). Decay times of surface plasmon excitation in metal nanoparticles by persistent spectral hole burning. Phys. Rev. Lett..

[44] Bosbach J., Hendrich C., Stietz F., Vartanyan T., Träger F. (2002). Ultrafast dephasing of surface plasmon excitation in silver nanoparticles: influence of particle size, shape, and chemical surrounding. Phys. Rev. Lett..

[45] SCHOTT Glass Database Number 517642.251. https://www.schott.com/d/advanced_optics/ac85c64c-60a0-4113-a9df-23ee1be20428/1.17/schott-optical-glass-collection-datasheets-english-may-2019.pdf.

[46] Zundel L., Manjavacas A. (2018). Finite-size effects on periodic arrays of nanostructures. J. Phys. Photonics.

[47] Sung J., Hicks E.M., Van Duyne R.P., Spears K.G. (2008). Nanoparticle spectroscopy: Plasmon coupling in finite-sized two-dimensional arrays of cylindrical silver nanoparticles. J. Phys. Chem. C.

[48] Matsushima A. Effect of periodicity in the light scattering from infinite and finite arrays of silver nanospheres. Proceedings of the 2017 IEEE International Conference on Computational Electromagnetics (ICCEM).

[49] Born M., Wolf E. (2013). Principles of Optics: Electromagnetic Theory of Propagation, Interference and Diffraction of Light.

[50] Shen H., Bienstman P., Maes B. (2009). Plasmonic absorption enhancement in organic solar cells with thin active layers. J. Appl. Phys..

[51] Starovoytov A.A., Nabiullina R.D., Gladskikh I.A., Polischuk V.A., Parfenov P.S., Kamalieva A.N. (2018). Plasmon-exciton interaction in the thin film of inhomogeneous ensemble of silver nanoparticles and cyanine J-aggregates. Nanophotonics VII.

